# Adjunctive empirical superior vena cava isolation with fourth-generation cryoballoon in paroxysmal atrial fibrillation does not improve outcomes and increases complications: the randomized CISPAF trial

**DOI:** 10.3389/fcvm.2026.1849859

**Published:** 2026-08-26

**Authors:** Vedran Pašara, Bruno Ban, Ivan Prepolec, Andrija Nekić, Zvonimir Katić, Domagoj Kardum, Davor Miličić, Gian-Battista Chierchia, Carlo De Asmundis, Vedran Velagić

**Affiliations:** 1 Department of Cardiovascular Diseases, University Hospital Centre Zagreb, Zagreb, Croatia; 2 Heart Rhythm Management Centre, Universitair Ziekenhuis Brussel, Postgraduate Program in Cardiac Electrophysiology and Pacing, Vrije Universiteit Brussel, European Reference Networks Guard-Heart, Brussels, Belgium; 3 School of Medicine, University of Zagreb, Zagreb, Croatia

**Keywords:** atrial fibrillation, catheter ablation, cryoballoon, pulmonary vein isolation, randomized trial, superior vena cava isolation

## Abstract

**Background:**

Pulmonary vein isolation (PVI) is the cornerstone of atrial fibrillation (AF) ablation, but non-pulmonary vein triggers, particularly the superior vena cava (SVC), may contribute to recurrence. Cryoballoon (CB) ablation has been applied for empirical SVC isolation (SVCI), yet evidence is limited and inconsistent.

**Objective:**

The CISPAF trial evaluated the feasibility, safety, and efficacy of adjunctive SVCI using a fourth-generation CB in patients undergoing first-time PVI for paroxysmal AF (PAF).

**Methods:**

CISPAF was a prospective, randomized, single-centre trial including 149 patients with PAF assigned to PVI alone (*n* = 74) or PVI + SVCI (*n* = 75). Ablations were performed with a fourth-generation 28-mm CB. The primary endpoint was 12-month freedom from AF; safety endpoints included a composite of major procedure-related complications, with particular attention to phrenic nerve injury (PNI) and sinus node impairment.

**Results:**

Acute PVI was achieved in all patients. SVCI succeeded in 62/75 (84.9%), with real-time isolation in 45 (60.0%). Major complications occurred only in the PVI + SVCI group (2.6% vs. 0%), including one sinus node injury requiring pacemaker implantation and one vascular complication requiring surgical repair. Transient or impending PNI and transient bradycardia/junctional rhythm were more frequent with PVI + SVCI (34.7% vs. 10.8% and 17.3% vs. 2.7%; respectively; both *p* < 0.01), but these events were self-limited and resolved after interruption of energy delivery. At 12 months, AF-free survival did not differ significantly between groups (89.3% vs. 79.7%; log-rank *p* = 0.096).

**Conclusion:**

Adjunctive SVCI using a fourth-generation CB did not demonstrate a statistically significant improvement in arrhythmia-free survival at one year and was associated with a higher incidence of procedure-related complications. These findings do not support the routine use of empirical SVCI during initial CB ablation for PAF and should be interpreted as hypothesis-generating.

## 1 Introduction

Atrial fibrillation (AF) is the most common sustained cardiac arrhythmia, associated with significant morbidity, mortality, and healthcare burden (1). Over the past three decades, pulmonary vein isolation (PVI) has become the established cornerstone of catheter ablation for AF (2). However, despite advances in techniques and technologies, mid- and long-term success rates remain suboptimal, and many patients continue to experience AF recurrence.

These limitations have led to ongoing efforts to refine ablation strategies by targeting arrhythmogenic sources beyond the pulmonary veins (PVs), which may initiate paroxysmal AF (PAF) in up to 28% of patients (3). Such sites include the superior vena cava (SVC), left atrial posterior wall, crista terminalis, coronary sinus ostium, ligament of Marshall, and interatrial septum (3–5). Among these, the SVC is recognized as a frequent source of ectopic activity, with triggers identified in 6–12.9% of patients undergoing ablation for PAF (4, 6–9).

Several non-randomized studies have suggested that empirical superior vena cava isolation (SVCI), when added to PVI, may improve ablation outcomes in patients with PAF (8, 10). In contrast, other studies found no significant reduction in AF recurrence with adjunctive SVCI (11, 12). Moreover, four randomized clinical trials (RCTs) of radiofrequency-based adjunctive SVCI have yielded inconsistent results, likely reflecting differences in patient populations and methodology (13–16). Some non-randomized studies utilizing cryoballoon (CB) for SVCI have shown encouraging results (17, 18), but a recent RCT employing a third-generation CB did not demonstrate improved outcomes, though it included both PAF and early persistent AF patients (19).

Consequently, given these conflicting findings, the clinical value of adjunctive SVCI remains uncertain and warrants further investigation. To address this gap, this study aimed to assess the impact of adjunctive SVCI using a fourth-generation CB in patients undergoing PVI for PAF.

## 2 Methods

### 2.1 Study design

**Figure 1:**
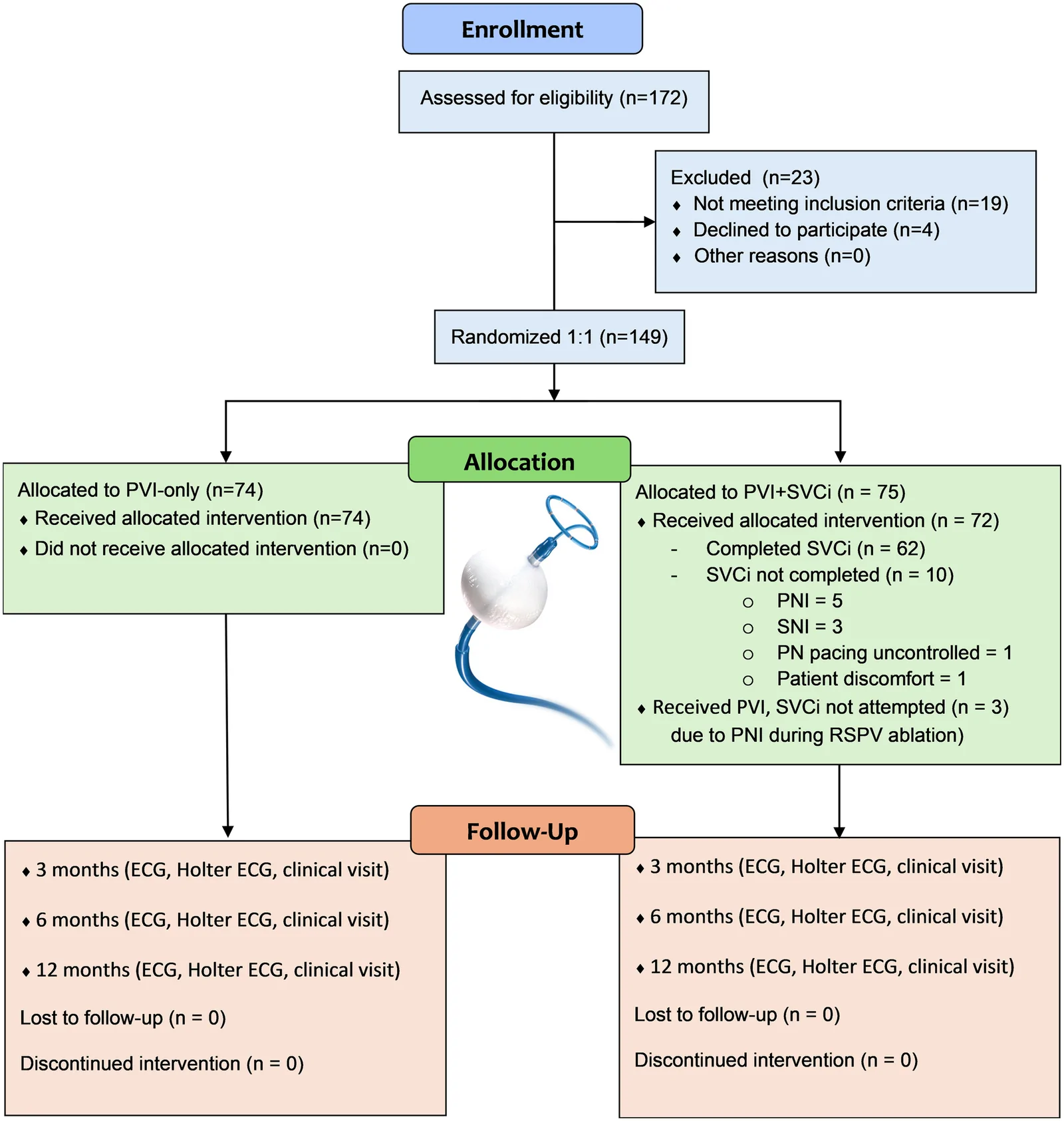
Flowchart of the trial*.* ECG, electrocardiogram; PN, phrenic nerve; PNI, phrenic nerve injury; PVI pulmonary vein isolation; RSPV, right superior pulmonary vein; SNI, sinus node impairment; SVCI, superior vena cava isolation.

The CISPAF trial (Cryoballoon Isolation of Superior Vena Cava in Paroxysmal Atrial Fibrillation) was a randomized, controlled, single-center cohort study designed to evaluate the safety and efficacy of adjunctive SVCI using a fourth-generation CB, in addition to standard PVI, in patients with PAF. We hypothesized that adjunctive SVCI would increase the efficacy and reduce AF recurrence compared to conventional ablation alone. The study flowchart is shown in Figure 1.

### 2.2 Study population

This study enrolled consecutive patients diagnosed with PAF who were referred for CB ablation. The clinical decision to treat AF invasively was made independently of this study protocol. Eligible patients were prospectively recruited and randomly assigned in a 1:1 ratio to either the intervention group, which received standard PVI plus empirical SVCI, or the control group, which underwent standard PVI alone.

Exclusion criteria were as follows: age <18 years; cognitive or communication barriers preventing informed consent; prior AF ablation; significant left atrial enlargement (diameter > 55 mm in the parasternal long-axis view); cardiomyopathy with manifest heart failure; unstable coronary artery disease; significant valvular heart disease; left atrial thrombus detected by preprocedural transesophageal echocardiography (TEE); severe renal impairment; and other significant non-cardiac comorbidities.

### 2.3 Randomization

The allocation sequence was generated using a computerized random number generator, employing simple randomization to ensure unbiased group assignment. Due to the single-center design and the relatively homogeneous patient population, no stratification by baseline characteristics such as age, sex, or comorbidities was performed. Allocation concealment was maintained by placing assignments in sequentially numbered, sealed, opaque envelopes, which were opened only after obtaining informed consent and immediately prior to the procedure. The operator was informed of the group assignment, while patients remained unaware of their assignment to maintain single-blind conditions.

### 2.4 Study size

At the time of study design, contemporary series of single-procedure cryoballoon PVI reported 1-year AF recurrence rates of approximately 20%–30%. Randomized data on empiric SVCI were limited and showed only a non-significant trend toward lower recurrence with adjunctive SVCI in PAF patients, without a robust or consistent effect size that could be used for a precise, clinically anchored power calculation (20). Therefore, this trial was conceived as an exploratory randomized study rather than being powered on a prespecified absolute risk reduction with SVCI. The sample size was determined to detect a medium effect size between two independent groups, using a two-tailed test with *α* (alpha) = 0.05% and 80% power. Calculations performed using G*Power software (version 3.1.9.4) indicated that a minimum of 128 participants (64 in each group) was required to achieve adequate power. To account for potential loss to follow-up and to further increase statistical power, additional participants were enrolled.

### 2.5 Ethical considerations

The study was approved by the Ethics Committee of the University Hospital Centre Zagreb and the Ethics Committee of the University of Zagreb School of Medicine. The protocol adhered to the ethical principles of the Declaration of Helsinki for medical research involving human subjects, ensuring participant privacy and data confidentiality. The study was registered at ClinicalTrials.gov (NCT05081310; 7 June 2021). All data were anonymized. Participants received clear information about the study and provided written informed consent before enrollment.

### 2.6 Preprocedural assessment

A transthoracic echocardiogram was performed within three months prior to ablation to evaluate left ventricular ejection fraction and rule out significant structural or valvular heart disease. All patients received uninterrupted anticoagulation therapy in line with current guidelines (21). For those on vitamin K antagonists, anticoagulation was maintained with a target international normalized ratio (INR) of 2.0–2.5. In patients treated with direct oral anticoagulants (DOACs), a minimally interrupted strategy was used: the morning dose on the day of the procedure was omitted, and DOAC therapy was resumed at the next scheduled dose, no earlier than four hours after ablation.

### 2.7 Ablation protocol

PVI was performed utilizing a 28-mm CB (Arctic Front Advance Pro™ with the Achieve Advance circular mapping catheter, Medtronic©, Minneapolis, MN, USA), following previously established protocols (22). Conscious sedation was achieved using fentanyl and midazolam.

Vascular access was obtained via two punctures of the right femoral vein. A 12 Fr sheath (FlexCath Advance, Medtronic Inc., Minneapolis, MN, USA) was used for the CB, while a 10 Fr sheath was used for the intracardiac echocardiography (ICE) catheter (AcuNav Acuson, Siemens AG, Germany). ICE imaging was used to visualize the left atrial appendage and to assess PV anatomy before being replaced by a decapolar catheter (Biotronik SE, Germany) for phrenic nerve (PN) pacing.

Transseptal puncture was performed under combined fluoroscopic and ICE guidance using a BRK-1 needle (St. Jude Medical, Minneapolis, MN, USA) and an 8.5 Fr long sheath (Swartz, Abbott, Chicago, IL, USA). Unfractionated heparin was administered immediately post-transseptal access to maintain an activated clotting time (ACT) > 300 s, with repeated ACT checks and supplemental boluses every 15–20 min.

PVI was carried out using a fourth-generation 28-mm CB (Arctic Front Advance Pro™, Medtronic©, Minneapolis, MN, USA) and a 20-mm octapolar circular mapping catheter (Achieve Advance™, Medtronic©, Minneapolis, MN, USA), which was positioned at each PV ostium to record baseline potentials. The CB was advanced to the PV antrum or ostium using an “over-the-wire” technique, and vein occlusion was confirmed by contrast injection showing complete contrast retention. PVs were targeted sequentially in a clockwise fashion: left superior, left inferior, right inferior, and right superior. If isolation was delayed (>60 s) or unsuccessful, the balloon was repositioned and a second application delivered. In cases where PV potentials were not recorded in real time, a second 180-second freeze was applied if the temperature had not reached −40 °C within the initial 60 s (23). If isolation could not be confirmed during application, the mapping catheter was retracted to a more proximal position within the PV ostium where signals had been documented before ablation (24). For common PV ostia, each branch was targeted separately with one cryoapplication per branch.

PN function was monitored during right-sided PV ablation by pacing the ipsilateral PN with a decapolar catheter in the right subclavian vein (cycle length 1,500 ms, 20 mA). Phrenic capture was confirmed both by manual palpation of diaphragmatic contractions and by observing corresponding fluctuations in central venous pressure (25). PN pacing began once the CB reached −20 °C to minimize the risk of balloon displacement. Cryoapplication was promptly terminated at any indication of PN compromise. PVI success was defined as elimination or dissociation of PV potentials on the circular mapping catheter. Adenosine testing for dormant conduction was not performed, consistent with current guidelines and its limited role in CB ablation (21).

**Figure 2:**
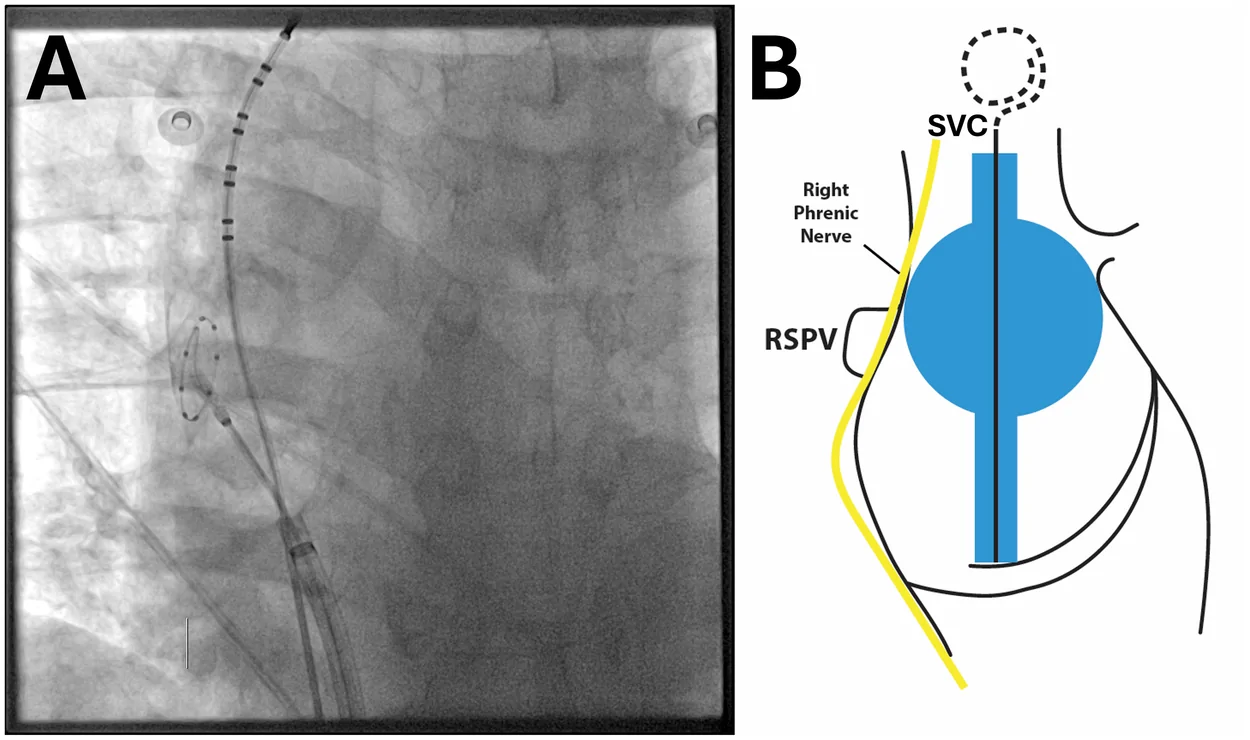
Superior vena cava (SVC) ablation in the posteroanterior view. **(A)** Fluoroscopic image showing a decapolar catheter in the right subclavian vein for ipsilateral phrenic nerve pacing, an Achieve Advance circular mapping catheter in the SVC, and an inflated cryoballoon positioned at the SVC ostium. **(B)** Schematic illustration of the close anatomical relationship between the SVC and the right phrenic nerve. RSPV, right superior pulmonary vein; SVC, superior vena cava.

After standard PVI, empirical SVC isolation was performed as described in prior studies (17, 18, 26). The CB was repositioned into the right atrium, with the circular mapping catheter advanced into the SVC. The balloon was inflated in the right atrium and moved towards the SVC ostium to achieve occlusion, confirmed by contrast injection (Figure 2). A single cryoapplication of 120–180 s was delivered. If SVC potentials and TTI were recorded, ablation was continued for 120 s post-isolation. PN function during SVC ablation was monitored by ipsilateral PN pacing (cycle length 1,200 ms, 20 mA), with both tactile and central venous pressure monitoring, allowing early detection of transient PN impairment and prompt cessation of energy delivery to reduce the risk of permanent damage. Groin access was closed using a figure-of-eight suture without the administration of protamine (27).

### 2.8 Post-procedural management and follow-up

All patients received standard post-procedural care after PVI. They were maintained in a supine position until the following morning and continuously monitored via telemetry throughout their hospital stay. Discharge typically occurred the day after the procedure. Before discharge, a transthoracic echocardiogram was performed to exclude any periprocedural complications.

Oral anticoagulation was continued for at least two months in all patients, with further continuation or discontinuation guided by individual thromboembolic risk assessed using the CHA₂DS₂-VASc score. Proton pump inhibitors were prescribed for six weeks to reduce the risk of esophageal complications.

A three-month blanking period was observed post-ablation. During this period, antiarrhythmic drugs (AADs) were initiated at the discretion of the treating physician, with discontinuation typically advised at the three-month follow-up. However, continuation or reinitiation of AADs beyond this period remained at the physician's discretion.

Outpatient follow-up visits were scheduled at 3, 6, and 12 months after the procedure, each including a clinical visit, 12-lead electrocardiogram (ECG), and 24-hour Holter ECG monitoring. In addition to scheduled follow-up, patients were instructed to obtain ECG recordings in case of symptoms suggestive of AF recurrence, allowing detection of symptomatic episodes between visits. In cases of AF recurrence, repeated ablation was considered. All redo procedures were performed using radiofrequency energy.

### 2.9 Study endpoints

The primary efficacy endpoint was freedom from documented AF at a one-year follow-up after a 3-month blanking period, defined as the absence of AF episodes lasting 30 s or longer on 12-lead ECG or 24-hour Holter monitoring. The primary safety endpoint was the incidence of major procedure-related complications (pericardial tamponade, stroke or TIA, persistent PNI at discharge, significant vascular complications requiring intervention, need for permanent pacemaker implantation, or death). Transient or impending PNI and transient sinus node impairment (SNI) (bradycardia or junctional rhythm resolving after termination of energy delivery) were recorded as procedural events rather than complications.

Secondary endpoints included acute procedural success, defined as complete PVI in all targeted veins and, in the PVI + SVCI group, acute SVCI; freedom from atrial arrhythmias (AF, AFL, AT) at one year, need for repeat ablation, total procedure duration, fluoroscopy time and radiation dose, as well as the number of cryoenergy applications required per PV or SVC.

### 2.10. Statistical analysis

Categorical data are presented as absolute and relative frequencies. Differences between categorical variables were assessed using the Chi-square test. Continuous variables were assessed for normality using the Shapiro–Wilk test. Due to the non-normal distribution of most continuous variables, they are reported as median with interquartile range (IQR). Differences in continuous variables between the two independent groups were assessed using the Mann–Whitney U test. Survival analysis was performed using the Kaplan–Meier method, and groups were compared with the log-rank test. The primary efficacy analysis was conducted according to the intention-to-treat principle, with patients analyzed in their randomized groups. All *P* values are two-tailed, and a *P* value of < 0.05 was considered statistically significant. Statistical analysis was performed using MedCalc® Statistical Software version 23.2.1 (MedCalc Software Ltd, Ostend, Belgium; https://www.medcalc.org; 2025).

## 3 Results

### 3.1 Patient characteristics

Between May 2021 and October 2023, a total of 149 patients were enrolled in the study, of whom 93 (62.4%) were men. The median age was 63 years (IQR, 56–70), and the median CHA2DS2-VASc score was 2 (IQR, 1–3). All participants had PAF, with a median interval from diagnosis to ablation of 18 months (IQR, 6–48). Patients were randomized to undergo either PVI alone (*n* = 74) or PVI with additional SVCI (*n* = 75). Baseline characteristics were comparable between groups and are detailed in Table 1.

**Table 1:** Baseline characteristics.

Characteristic	All (*n* = 149)	PVI-only (*n* = 74)	PVI + SVCI (*n* = 75)	*p* value*
Age, y^b^	63 (56–70)	63 (55–70)	63 (58–70)	0.99
Male, N (%)	93 (62.4)	45 (60.8)	48 (64)	0.31^a^
BMI, kg/m^2^^b^	27.9 (24.8–31.2)	27.6 (24.2–30.4)	29.3 (24.5–31.8)	0.19
CHA2DS2-VASc score^b^	2 (1–3)	2 (1–3)	2 (1–3)	0.84
AF history, mo^b^	18 (6–48)	18 (6–48)	16 (6–45)	0.06
Arterial hypertension, N (%)	122 (81.9)	59 (79.7)	63 (84)	1.0^a^
Diabetes mellitus, N (%)	24 (16.1)	10 (13.5)	14 (18.7)	0.51^a^
Dyslipidemia, N (%)	86 (57.7)	41 (55.4)	45 (60)	0.41^a^
CKD, N (%)	10 (6.7)	4 (5,4)	6 (8)	0.75^a^
COPD, N (%)	4 (2.7)	1 (1.4)	3 (4)	1.0^a^
OSAS, N (%)	4 (2.7)	1 (1.4)	3 (4)	1.0^a^
CAD, N (%)	11 (7.4)	3 (4,1)	8 (10,7)	1.0^a^
TIA/CVA, N (%)	6 (4)	2 (2.7)	4 (5.3)	0.44^a^
Smoking, N (%)	32 (21.5)	14 (18.9)	18 (24)	0.44^a^
LVEF, %^b^	60 (60–65)	60 (60–65)	60 (60–65)	0.46
LAd, mm^b^	39 (33.8–43.3)	39 (34–43.2)	41.5 (36–44.5)	0.85
LAVi, mL/m^2^^b^	32.5 (26–40.4)	32 (27–38)	33.8 (25.9–40.0)	0.34
Class I AAD, *n* (%)	47 (31.5)	23 (31.1)	24 (32)	0.80^a^
Class II AAD, *n* (%)	101 (67.8)	45 (60.8)	56 (74.7)	0.36^a^
Class III AAD, *n* (%)	40 (26.8)	16 (21.6)	24 (32)	0.66^a^
DOAC, *n* (%)	142 (95.3)	69 (93.2)	73 (97.3)	1.0^a^
ACE-I/ARB, *n* (%)	93 (62.4)	45 (60.8)	48 (64)	1.0^a^

a Fisher's exact test.

b Continuous data are presented as median and interquartile range.

*Mann–Whitney U test.

AAD, antiarrhythmic drug; ACE-I, angiotensin-converting enzyme inhibitor; AF, atrial fibrillation; ARB, angiotensin receptor blocker; BMI, body mass index; CKD, chronic kidney disease; COPD, chronic obstructive pulmonary disease; CVA, cardiovascular accident; LAd, left atrium diameter; LAVI, left atrium volume index; LVEF, left ventricular ejection fraction; PVI, pulmonary vein isolation; SVCI, superior vena cava isolation; TIA, transient ischemic attack.

### 3.2 Procedural characteristics

The median procedure duration was 54 min (IQR, 45–65), with a median fluoroscopy time of 6 min (IQR, 4–9), a median total radiation dose of 9 mGy (IQR, 4–38), and a median contrast volume of 20 mL (IQR, 15–40). There were no significant differences in these procedural parameters between the groups, as shown in Table 2.

**Table 2:** Procedural characteristics.

Characteristic	All (*n* = 149)	PVI-only (*n* = 74)	PVI + SVCI (*n* = 75)	*p* value*
Procedure time, min	54 (45–65)	55 (44–65)	53 (45–65)	0.93
Fluoroscopy time, min	6 (4–9)	6 (3.9–9.6)	5 (4.0–8.0)	0.68
Total radiation dose, mGy	9 (4–38)	12 (4–51,2)	6 (3,8–22)	0.66
Contrast volume, mL	20 (15–40)	20 (15–40)	22.5 (20–35)	0.70

*Mann–Whitney U test; Continuous data are presented as median and interquartile range.

PVI, pulmonary vein isolation; SVCI, superior vena cava isolation.

At the time of ablation, 66 patients (89.2%) in the PVI-only group and 72 patients (96%) in the PVI + SVCI group were in sinus rhythm, while 8 patients (10.8%) and 3 patients (4%), respectively, were in AF. During the procedure, 4 patients (5.4%) in the PVI-only group and 2 patients (2.6%) in the PVI + SVCI group converted to sinus rhythm as a direct result of ablation. The remaining patients who were still in AF underwent electrical cardioversion at the end of the procedure: 4 patients (5.4%) in the PVI-only group and 1 patient (1.3%) in the PVI + SVCI group. There were no statistically significant differences between the groups in baseline rhythm status (*p* = 0.13) or conversion outcomes (*p* = 0.36).

### 3.3 Acute ablation outcomes

#### 3.3.1 Pulmonary vein ablation outcomes

Acute PVI was accomplished in all patients in both study arms. In the PVI-only group, 13 left common pulmonary trunks were isolated, while in the PVI + SVCI group, 14 left and one right common pulmonary trunk were isolated. Detailed ablation characteristics for each PV are presented in Table 3. There were no significant differences between the groups in acute isolation rates, number of applications, or procedural temperatures.

**Table 3:** Ablation characteristics.

Characteristic	PVI-only (*n* = 74)	PVI + SVCI (*n* = 75)	*p* value
*Left superior pulmonary vein*			
Cryoapplications, n	1 (1–1)	1 (1–1)	0.46
Application time, s	180 (180–240)	180 (180–240)	0.92
Time to isolation, s	38.5 (29.8–51.3)	39 (28.5–52.5)	0.99
Temperature at isolation, °C	−35 (−40–−30)	−37 (−41–−25)	0.87
Nadir temperature, °C	−52 (−55–−46,3)	−51 (−52,5–−46,5)	0.12
Thaw time, s	11 (9–14,3)	10 (8–12)	**0**.**03**
Time to rewarming +20 °C, s	55 (45–61)	50 (38,8–55)	0.14
*Left inferior pulmonary vein*			
Cryoapplications, n	1 (1–1)	1 (1–1)	0.49
Application time, s	240 (190–240)	240 (180–240)	0.11
Time to isolation, s	30 (22.5–41.5)	27 (22–38)	0.38
Temperature at isolation, °C	−30 (−35.3–−21.5)	−28 (−33–−19)	0.31
Nadir temperature, °C	−45 (−50–−43)	−46 (−48.3–−43)	0.93
Thaw time, s	9 (7–10,5)	8 (7–10)	0.56
Time to rewarming +20 °C, s	40 (33–50)	40 (29.5–50)	0.93
*Left common pulmonary vein*			
Cryoapplications, n	2 (2–2)	2 (2–3)	0.24
Application time, s	420 (270–480)	450 (420–675)	0.11
Time to isolation, s	52 (40–76)	53 (34,5–80)	0.74
Temperature at isolation, °C	−44 (−48.5–−37)	−38 (−41.8–−30.5)	0.14
Nadir temperature, °C	−56 (−60.5–−49.5)	−46 (−52,3–−42)	**0**.**01**
Thaw time, s	13.5 (9–16.5)	9 (6.8–12.3)	**0**.**03**
Time to rewarming +20 °C, s	47.5 (32.8–75)	52.5 (28.8–62.5)	0.67
*Right superior pulmonary vein*			
Cryoapplications, n	1 (1–1)	1 (1–1)	0.53
Application time, s	180 (180–240)	180 (180–240)	0.70
Time to isolation, s	31 (22–46)	30 (19–43)	0.35
Temperature at isolation, °C	−34 (−41–−25)	−31 (−38–−20)	0.15
Nadir temperature, °C	−53 (−56–−48)	−51,5 (−55–−46)	**0**.**04**
Thaw time, s	10 (8–13)	9 (7–12.3)	0.36
Time to rewarming +20 °C, s	52 (32.5–60)	50 (35–61)	0.80
*Right inferior pulmonary vein*			
Cryoapplications, n	1 (1–1)	1 (1–1)	0.92
Application time, s	180 (180–240)	180 (180–240)	0.79
Time to isolation, s	37 (27–48)	40 (28–53.8)	0.43
Temperature at isolation, °C	−35 (−38–−30)	−36 (−41–−29.8)	0.36
Nadir temperature, °C	−47 (−52–−43)	−47 (−53–−44)	0.64
Thaw time, s	9 (7–10)	8 (6–10)	0.29
Time to rewarming +20 °C, s	39.5 (30–54.5)	50 (25–57.5)	0.50
*Superior vena cava*			
Cryoapplications, n	-	1 (1–1)	N/A
Application time, s	-	131.5 (92–179)	N/A
Time to isolation, s	-	22 (15–52,5)	N/A
Temperature at isolation, °C	-	−30.5 (−37.8–−18)	N/A
Nadir temperature, °C	-	−48 (−55–−41)	N/A
Thaw time, s	-	7.5 (5–11.8)	N/A
Time to rewarming +20 °C, s	-	25 (15.3–42.5)	N/A

*Mann–Whitney U test; Continuous data are presented as median and interquartile range.

Bold values denote statistical significance.

N/A, not applicable.

#### 3.3.2 Superior vena cava ablation outcomes

Successful SVCI was achieved in 62 patients (84.9%) in the PVI + SVCI group. Notably, SVCI could not be achieved in approximately 15% of patients, primarily due to safety-related interruptions. The primary reasons for early interruption of SVC ablation were signs of impending right PNI (5/10), onset of SNI (3/10), inability to maintain PN capture during pacing (1/10) or patient-reported discomfort (1/10). In addition, three patients in the PVI + SVCI group did not undergo SVC ablation due to PNI detected during right PV ablation.

**Figure 3:**
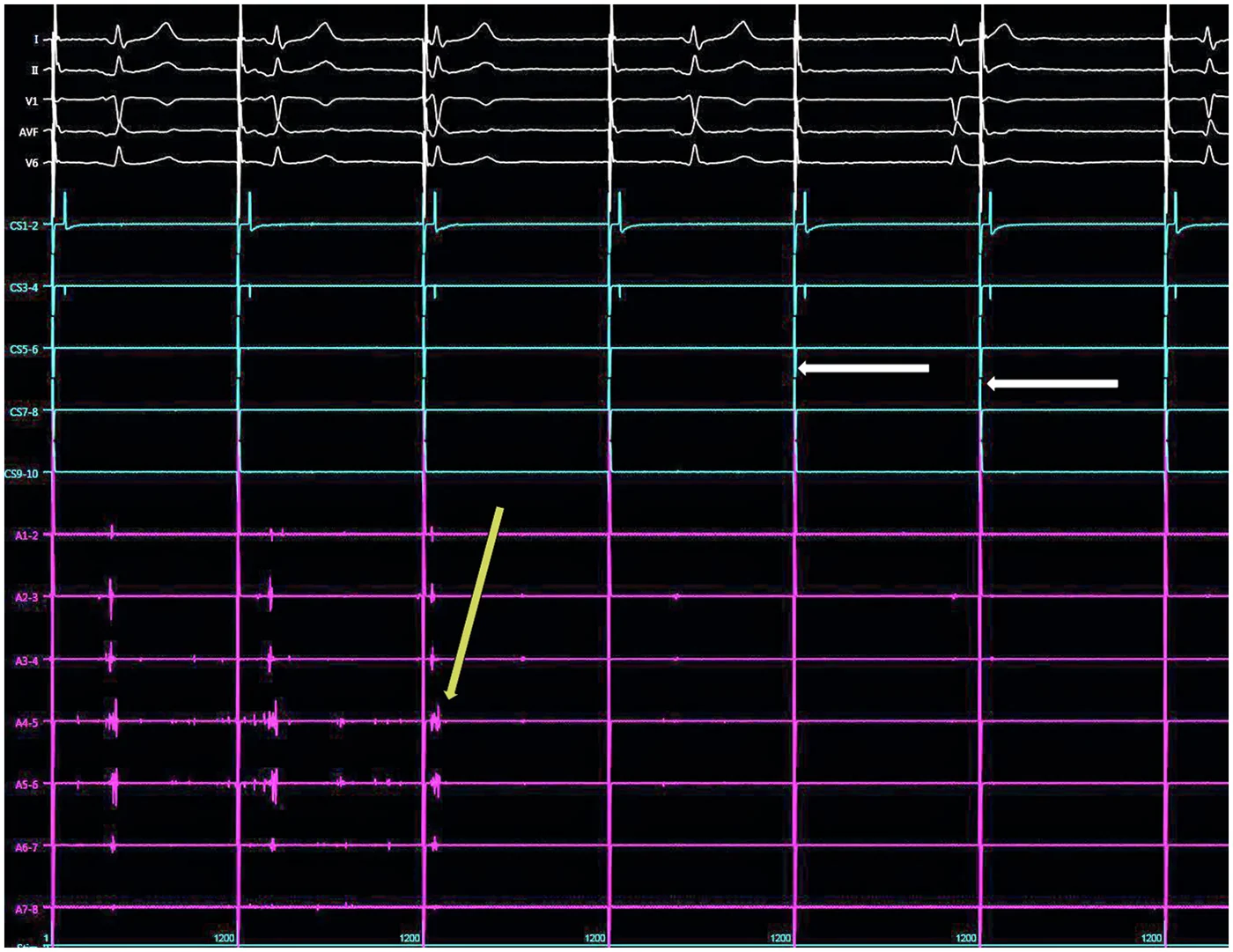
Real-time SVC isolation. Example of potentials recorded at the SVC ostium during cryoapplication in sinus rhythm, showing baseline activity prior to isolation and subsequent electrical isolation of the SVC. The yellow arrow marks the last SVC signal recorded by the circular mapping catheter before isolation. Surface leads I, II, aVF, V1, and V6, as well as bipolar intracardiac electrograms from the Achieve Advance circular mapping catheter (A1–2 to A7–8), are displayed. A decapolar catheter positioned in the subclavian vein was used for ipsilateral phrenic nerve pacing, with pacing spikes visible (white arrows).

The intended 180-second cryoapplication in the SVC was successfully completed in 18 patients (24%), while 48 patients (64%) received applications lasting at least a 120-seconds. In most cases, cryoenergy delivery was stopped prematurely due to signs of right PNI, SNI, or patient-reported discomfort. The median time to achieve SVC isolation was 22 s (IQR, 15–52.5), with a median temperature at isolation of −30.5 °C (IQR, −37.8 to −18). At 60 s, the median temperature was −43 °C (IQR, −48 to −36), and the lowest temperature reached during ablation was −48 °C (IQR, −55 to −41). Real-time recordings of SVC isolation were documented in 45 patients (60%) (Figure 3). Additional ablation characteristics are provided in Table 3.

### 3.4 Safety outcomes

Major complications were infrequent and did not differ significantly between groups (0% vs. 2.6%; *p* = 1.0, Fisher's exact test). Nonetheless, two major adverse events were recorded during the study: one patient in the PVI + SVCI group required permanent AAI pacemaker implantation due to SNI, and another developed a significant vascular complication involving both a pseudoaneurysm and an arteriovenous fistula, which necessitated surgical repair.

Impending or transient PNI occurred in 34 patients (22.8%): 8 patients (10.8%) in the PVI-only group and 26 patients (34.7%) in the PVI + SVCI group (*p* = 0.001, Fisher's exact test). Among the affected patients in the PVI + SVCI group, 24 (82.8%) experienced impending or transient PNI specifically during SVC ablation. In all cases, discontinuation of cryoenergy delivery led to complete recovery of PN function by the end of the procedure, and no cases of persistent PNI were observed. Bradycardia or junctional rhythm, which may reflect either vagotonia or acute SNI during SVCI, occurred in 13 patients (17.3%) in the PVI + SVCI group, and in 2 patients (2.7%) in the PVI-only group (*p* = 0.005, Fisher's exact test). Minor vascular complications, specifically groin hematomas, were reported in two patients (1.3%). Additionally, two cases of transitory gastroparesis occurred, both of which resolved with conservative treatment. No pericardial complications or cerebrovascular events occurred in either group. Complications are detailed in Table 4.

**Table 4:** Complications.

Characteristic		All (*n* = 149)	PVI-only (*n* = 74)	PVI + SVCI (*n* = 75)	*p* value*
PNI	Overall, *n* (%)	34 (22.8)	8 (10.8)	26 (34.7)	**0**.**001**
	Impending, *n* (%)	18 (12.1)	3 (4.1)	15 (20)	**0**.**005**
	Transitory, *n* (%)	16 (10.7)	5 (6.8)	11 (14.7)	0.19
	During right PVI, *n* (%)	10 (6.7)	5 (6.8)	5 (6.7)	1.0
	During SVCI, *n* (%)	-	-	24 (32)	N/A
Bradycardia or junctional rhythm, *n* (%)		15 (10.1)	2 (2.7)	13 (17.3)	**0.005**
Femoral hematoma, *n* (%)		3 (2)	1 (1.4)	2 (2.7)	1.0
Femoral pseudoaneurysm, *n* (%)		1 (0.7)	0 (0)	1 (1.3)	1.0
Femoral AV fistula, *n* (%)		1 (0.7)	0 (0)	1 (1.3)	1.0
Gastroparesis, *n* (%)		2 (1.3)	0 (0)	2 (2.7)	0.49

*Fisher's exact test.

Bold values denote statistical significance.

N/A, not applicable; PNI, phrenic nerve injury; PVI, pulmonary vein isolation; SVCI, superior vena cava isolation.

### 3.5 Mid-term clinical outcomes

Early recurrences of AF were observed in 16 patients (10.7%) during the blanking period: 9 patients (12.2%) in PVI-only group and 7 patients (9.3%) in PVI + SVCI group (*p* = 0.58, Fisher's exact test). In accordance with current guideline recommendations, these episodes were not considered treatment failures and were excluded from the primary endpoint analysis. Post-blanking AAD use did not differ significantly between groups (35.1% vs. 28.0%; *p* = 0.35).

**Figure 4:**
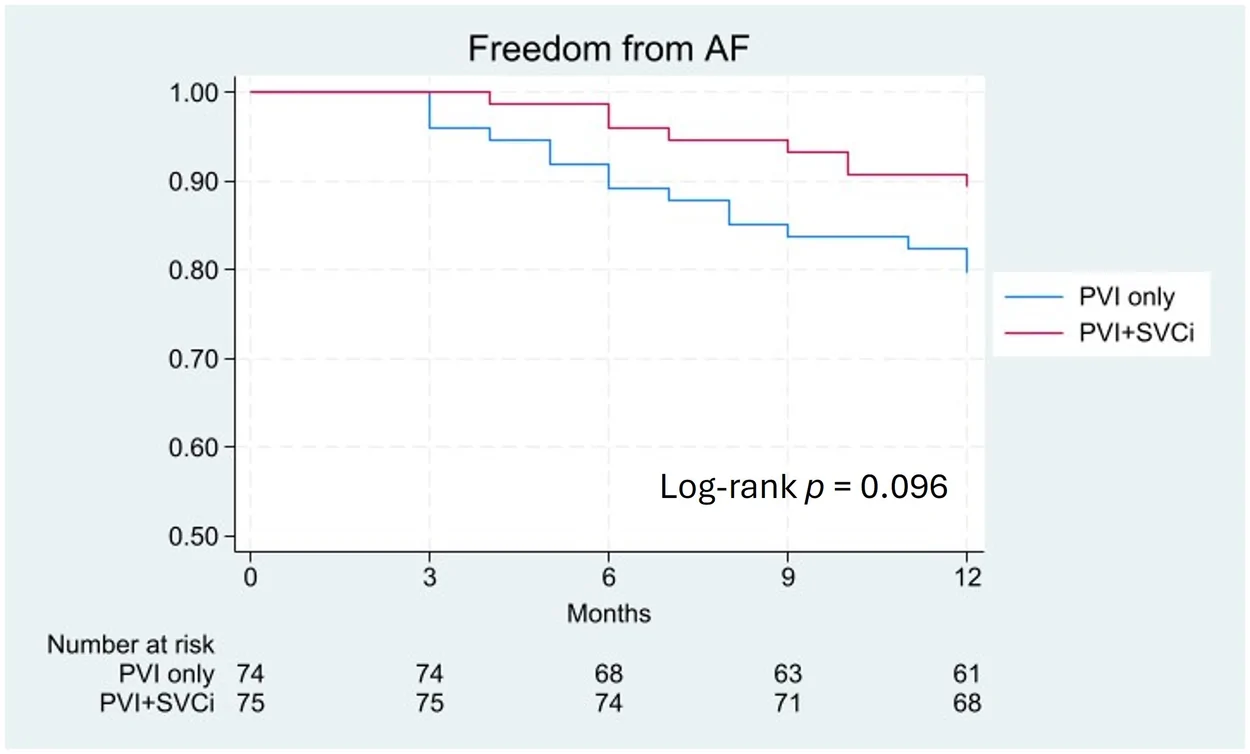
Freedom from AF by Kaplan–Meier analysis in the PVI-only and PVI + SVCI groups. PVI, pulmonary vein isolation; SVCI, superior vena cava isolation.

The primary clinical endpoint, freedom from AF at the 12-month follow-up, was 84.6% (95% CI: 78.7–90.5%) in the whole cohort, with a median time to AF recurrence of 6.5 (IQR: 4–9) months. There was no significant difference in freedom from AF between the PVI + SVCI and PVI-only groups (89.3%, 95% CI: 82.2–96.4% vs. 79.7%, 95% CI: 70.5–88.9%; log-rank *p* = 0.096) (Figure 4). The hazard ratio for AF recurrence with PVI + SVCI was 0.49 (95% CI 0.21–1.16, *p* = 0.11), suggesting a numerically lower recurrence rate that did not reach statistical significance.

During the 12-month follow-up, six patients (3.8% of the cohort) experienced atrial flutter (AFL), with three cases (3.6%) in the PVI + SVCI group and three (4.0%) in the PVI-only group (*p* = 0.653, Fisher's exact test). All episodes were classified as typical AFL. Freedom from any atrial arrhythmia (AF/AFL/atrial tachycardia) at the 12-month follow-up was 81.2% overall (95% CI: 74.9–87.5%), with 84.0% (95% CI: 75.8–92.2%) in the PVI + SVCI group compared to 78.4% (95% CI: 69.0–87.8%) in the PVI-only group (log-rank *p* = 0.37; HR = 0.71, 95% CI: 0.34–1.50, *p* = 0.37).

### 3.6 Redo procedures

**Figure 5:**
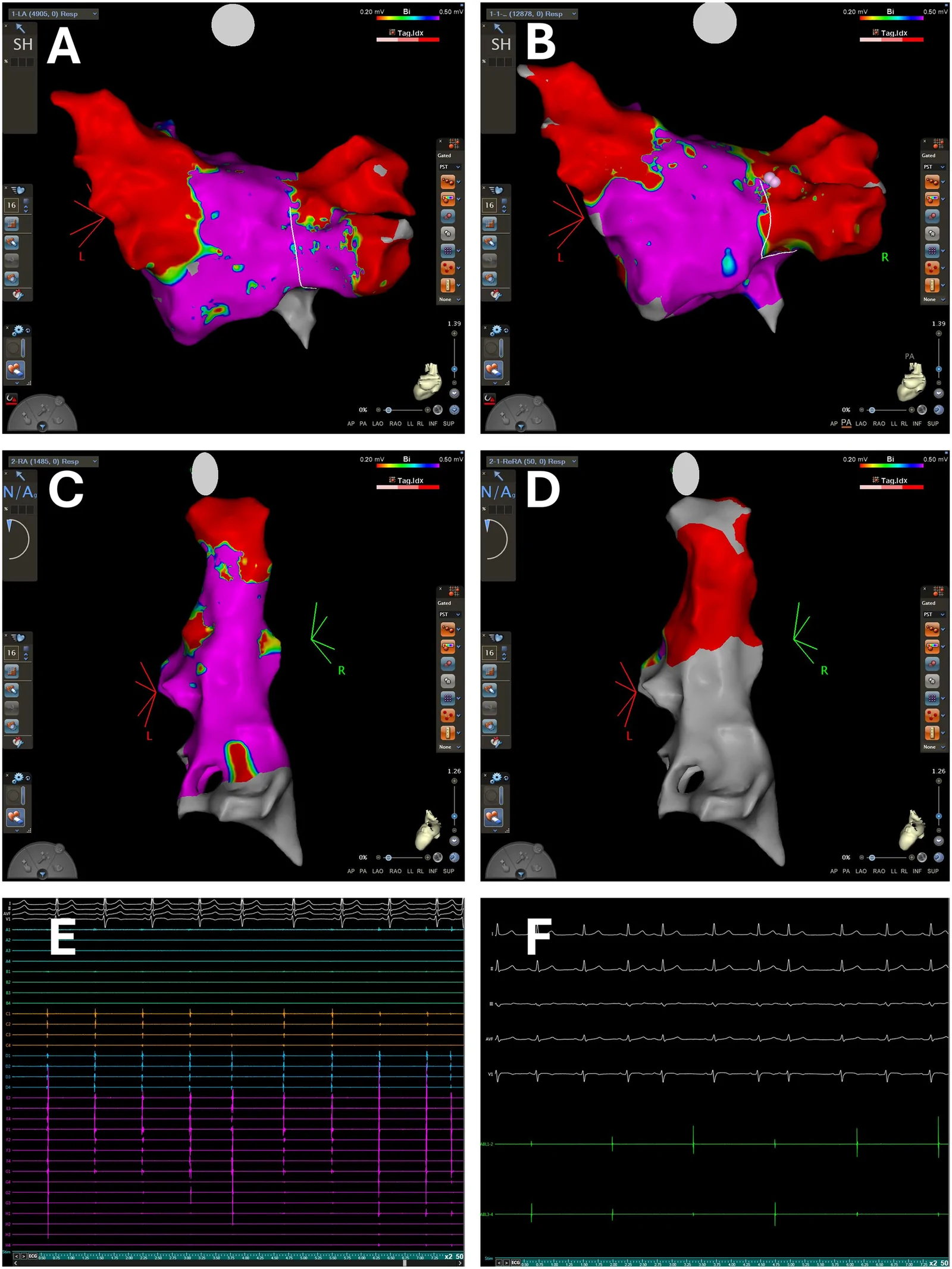
Redo procedure with the CARTO system in a patient in whom SVC cryoablation was interrupted after 120 s due to impending phrenic nerve injury, without real-time isolation. **(A)** Pre-ablation map of the left atrium showing reconnection of the right inferior pulmonary vein. **(B)** Post-ablation map of the left atrium showing all pulmonary veins isolated. **(C)** Pre-ablation map showing non-isolated SVC. **(D)** Post-ablation map showing isolated SVC. **(E)** Pre-ablation SVC potentials recorded with the Octaray mapping catheter. **(F)** Post-ablation dissociated SVC potentials recorded with the ablation catheter. SVC, superior vena cava.

Among 23 patients with AF recurrence, 4 (17.4%) underwent redo procedures during the 12-month follow-up: 3 in the PVI-only group versus 1 in the PVI + SVCI group (*p* = 0.37, Fisher's exact test) (Figure 5). The remaining patients either declined or were scheduled for future redo ablation. All redo procedures utilized radiofrequency ablation and were performed at a median of 8.5 months (IQR: 6.25–10.75) after the initial procedure. The mean number of reconnected veins per procedure was 1.0 ± 0.8 per procedure. No complications were reported during redo procedures.

## 4 Discussion

The CISPAF randomized controlled trial evaluated the effect of adding SVCI with a fourth-generation CB to standard PVI on AF recurrence in patients with PAF. The main findings of the study are as follows: (a) acute SVCI using a fourth-generation CB was achieved in the majority of patients in the intervention group; (b) the addition of SVCI was associated with a higher incidence of procedure-related events, particularly transient PNI and bradyarrhythmias, while (c) major complication rates remained low and similar across both groups; (d) adjunctive SVCI did not demonstrate a statistically significant improvement in arrhythmia-free survival; (e) the addition of SVCI did not result in a significant increase in procedural or fluoroscopy times compared to PVI alone.

CB ablation has become an established modality for PVI in the management of AF over the past two decades (28). Advances in CB technology have enabled more consistent lesion formation and expanded its use to certain non-pulmonary vein targets, such as the SVC (29). Accordingly, CB ablation has been employed for SVCI, demonstrating feasibility and high procedural efficacy, with acute success rates ranging from 80.8 to 93.8% in studies using second- and third-generation CBs (17, 19, 30). The acute success rate in our study was consistent with these findings, at 84.9%.

Despite acceptable acute success rates, concerns remain regarding safety issues, particularly the risk of collateral injury to the right PN and the sinus node (31). In this context, episodes of bradycardia or junctional rhythm observed during SVCI can be regarded as surrogate indicators of acute SNI (32, 33), although such findings in the PVI-only group are unlikely to be sinus node–related and may instead reflect transient autonomic effects (34). In our study, procedural events arising from the close anatomic proximity of these structures to the SVC represented the principal limitation to achieving complete SVCI, necessitating vigilant procedural monitoring and frequently leading to premature termination of ablation, thereby often impeding adequate delivery of cryoablation energy to the SVC with the 28-mm CB. Importantly, SVCI could not be achieved in approximately 15% of patients, largely due to safety-related interruptions, underscoring the limited practicality of this approach. Although several studies using CB ablation for PVI with adjunctive SVCI have reported low overall complication rates comparable to conventional CB PVI, with PNI incidences of up to 5% (17, 35–37), we observed a significantly higher rate of transient PNI and transient bradycardia/junctional rhythms consistent with the following reports. Wei et al. reported transient PNI in 19.2% of cases and SNI in 7.7% when using a second-generation CB (30). Similarly, the recent CAVAC AF trial using a third-generation CB reported PNI in 13.3% (predominantly in patients undergoing combined PVI and SVCI) and SNI in 18.8% (19). Our higher rate of these procedural events likely reflects rigorous real-time phrenic nerve and sinus node monitoring, enabling detection of transient but reversible events that may be underreported elsewhere. Importantly, the high incidence of PNI observed during SVC ablation highlights the narrow safety margin of this approach, even in experienced hands and despite careful monitoring. Although the incidence of PNI in the PVI-only group of our study (10.8%) was higher than the 4.2% reported in the large multicenter YETI registry (38), all PNIs observed were transient and fully resolved by the end of the procedure, thereby limiting their long-term clinical impact. However, these findings suggest that, although SVCI with a CB is technically feasible, the combination of a large balloon footprint and the adjacent PN and sinus node region makes the SVC a particularly vulnerable site and challenges the practicality of empirical CB-based SVCI in routine practice. Furthermore, the incidence of major complications in our study was low across both groups and aligned with rates reported in recent registries (3.4%–4.7%) (39, 40).

The clinical benefit of adjunctive SVCI alongside standard PVI remains debatable. The meta-analysis by Sharma et al. (20), which included three radiofrequency-based RCTs (13–15), found that empiric SVCI does not reduce AF recurrence rates in the overall AF population, although a trend toward statistical significance was observed in the PAF subgroup, consistent with our findings. In contrast, a more recent meta-analysis by Mariani et al. (6), which included a study by Dong et al. (16) alongside the aforementioned trials, demonstrated a significant reduction in AF recurrence within the PAF population. Importantly, differences in ablation strategies and the relatively small sample sizes across these studies should be acknowledged as key limitations to the interpretations of these results. Several CB-based studies have reported favorable outcomes; nevertheless, their non-randomized design raises concerns regarding potential bias (17, 18, 30).

The recent CAVAC AF trial, which included both PAF and early persistent AF patients and employed a third-generation CB, did not demonstrate improved outcomes with adjunctive SVCI; however, the heterogenous patient population may have influenced its results (19). In comparison, our study showed relatively high rates of freedom from AF at 12 months in both groups, suggesting that both differences in patient selection and ablation technology may contribute to between-study variability. The relatively higher success rates observed in our cohort may be attributed to the exclusive enrollment of patients with PAF and the use of a fourth-generation CB, which has previously been shown to improve procedural efficiency and safety for PVI (41, 42). In contrast, rhythm monitoring in the CAVAC AF trial was more rigorous, with daily ECG recordings, which may partly explain the observed discrepancy, as more intensive monitoring often reveals lower efficacy outcomes (43).

Although the point estimate of the hazard ratio for AF recurrence favored the PVI + SVCI group, this difference did not reach statistical significance. Given that our trial was conceived as an exploratory randomized study and was not powered on a prespecified absolute risk reduction for the primary clinical endpoint, these efficacy findings should be interpreted with caution. The present results therefore do not provide statistically robust evidence that adjunctive empirical SVCI improves arrhythmia-free survival, and they do not support its routine use in this setting; however, they also cannot definitively exclude a modest benefit in larger or differently selected populations.

The primary efficacy analysis in this trial was conducted according to the intention-to-treat principle, with all patients analyzed in their randomized groups. Notably, SVCI could not be completed in approximately 15% of patients allocated to PVI + SVCI because of safety-related interruptions, and a small number did not undergo SVC ablation at all owing to phrenic nerve impairment during right PV ablation. This incomplete delivery of SVCI would be expected to dilute any true treatment effect and bias the intention-to-treat efficacy analysis toward the null, further reinforcing the need to interpret the non-significant difference in AF-free survival with caution.

Current evidence regarding fourth-generation CB technology for SVCI is limited, and, to the best of our knowledge, the CISPAF trial is the first RCT to employ this tool for SVCI and systematically evaluate its feasibility, safety, and outcomes. Procedural parameters were comparable between groups, suggesting that adjunctive SVCI has minimal impact on workflow or resource utilization. Notably, overall fluoroscopy time was substantially shorter in our study compared with CAVAC AF, likely reflecting the impact of a previously established radiation-reduction protocol (44).

In this context, the elevated risk of collateral tissue damage, combined with the absence of a substantial improvement in arrhythmia-free survival, argues against the routine empirical use of CB-based SVCI and further questions its practicality in routine clinical practice. Of note, the absence of a significant clinical benefit may be explained by the relatively low prevalence of clinically relevant SVC triggers in PAF, which limits the potential value of routine empirical SVCI but suggests that targeted SVCI may still be considered in carefully selected patients with documented SVC triggers (16) or recurrent arrhythmia despite durable PVI (45), particularly when procedural parameters such as energy source, application settings, and device selection are tailored to mitigate risk.

Recent data and early experience suggest that pulsed-field ablation (PFA) may overcome some limitations of thermal energy sources and enable efficient and safe SVCI with a very low risk of collateral injury (46), particularly when combined with ICE guidance (47). Although PFA is rapidly emerging as a non-thermal technology for AF ablation, CB PVI continues to be used extensively worldwide. In this context, our observations may help inform lesion-set selection in centers that still use CB technology and serve as a reference for future investigations evaluating SVCI with newer non-thermal energy sources.

## 5 Limitations

This study has several limitations. First, it was conducted at a single center with a relatively modest sample size, which may limit the generalizability of the findings. Although the trial was designed with adequate *a priori* power to detect a medium effect size, the observed between-group difference in AF-free survival was clinically relevant but not statistically significant, likely reflecting the limited sample size and number of events. Accordingly, the efficacy findings do not provide definitive evidence and should be interpreted as hypothesis-generating. In addition, randomization was not stratified by baseline risk factors, which, in a modest-sized trial, could allow chance imbalances in prognostic variables that might influence AF recurrence; however, baseline characteristics were generally well balanced between groups. Second, only patients with paroxysmal AF were enrolled; therefore, the results cannot be extrapolated to broader AF populations, such as those with persistent or long-standing persistent AF. Third, although follow-up included scheduled visits with ECG and 24-hour Holter monitoring, supplemented by symptom-driven ECGs, this approach may have led to under-detection of asymptomatic or short-lived recurrences and thus to overestimation of efficacy, with higher apparent freedom-from-AF rates compared with studies using continuous or more intensive monitoring. However, as this was an investigator-initiated trial without external funding, resource constraints precluded the use of continuous monitoring. This limitation applies to both randomized groups and is unlikely to have introduced a systematic bias favouring or disadvantaging SVCI, but it may partly explain the relatively high apparent AF-free survival rates observed in both arms. Fourth, the trial exclusively employed a fourth-generation CB, and the findings may not be applicable to other technologies or energy sources. Moreover, while procedures were performed by experienced operators, the generalizability of these results to centers with less experience using fourth-generation CB technology may be limited. Finally, the study was not powered to detect rare adverse events, and larger multicenter trials with longer follow-up will be necessary to better define the safety profile and long-term efficacy of adjunctive SVCI.

## 6 Conclusion

Adjunctive SVCI using a fourth-generation CB in patients undergoing first-time catheter ablation for PAF was feasible and achieved high acute success rates without increasing procedure or fluoroscopy times. However, this approach was associated with a higher burden of procedure-related adverse events and did not demonstrate a statistically significant improvement in arrhythmia-free survival. These findings do not support the routine use of adjunctive SVCI during initial ablation for PAF and should be interpreted as hypothesis-generating rather than as definitive evidence against any potential benefit of SVCI. while Its role may warrant further evaluation in the era of emerging non-thermal energy sources such as PFA.

## Data Availability

The raw data supporting the conclusions of this article will be made available by the authors, without undue reservation.
